# Multi-dimensional regulation of tea leaf development: morphogenesis, hormone–transcriptional networks, environmental factors, and artificial cultivation practices

**DOI:** 10.3389/fpls.2026.1792941

**Published:** 2026-03-30

**Authors:** Can Xu, Xiang Liu, Shuai Li, Sitian Fei, Jihui Tan, Fang Zhou, Kunbo Wang, Yong Luo

**Affiliations:** 1 School of Chemistry and Environmental Science, Xiangnan University, Chenzhou, Hunan, China; 2 Nanling Research Institute for Modern Seed Industry, Xiangnan University, Chenzhou, Hunan, China; 3 Xiangnan University-Chenzhou Industrial Technology Research Institute, Chenzhou, Hunan, China; 4 Chenzhou Institute of Agricultural Sciences, Chenzhou, Hunan, China; 5 Key Laboratory of Tea Science of Ministry of Education & Key Laboratory for Evaluation and Utilization of Gene Resources of Horticultural Crops, Ministry of Agriculture and Rural Affairs of China, Hunan Agricultural University, Changsha, Hunan, China; 6 Yuelushan Laboratory, Changsha, Hunan, China

**Keywords:** leaf development, mechanisms, morphogenesis, regulation, tea plant

## Abstract

Tea (*Camellia sinensis*) is an important cash crop worldwide, with leaf development directly determines the yield, quality and economic value of tea products. Tea leaf formation is mainly controlled by a four-dimensional integrated regulatory framework. Specifically, leaf morphogenesis provides the structural basis, endogenous hormone–transcription factor networks form the core regulatory hub, environmental signals (e.g., light, temperature, water, altitude, pests, and diseases) provide the external driving force, and artificial cultivation practices enable precise human intervention. These four dimensions synergistically modulate leaf morphological structures, physiological functions, and secondary metabolite accumulation throughout the developmental cycle. Despite recent advances in research on individual regulatory factors, the cross-talk between multi-dimensional factors and the core regulatory nodes affecting leaf development remain poorly elucidated. Moreover, a comprehensive review integrating multi-scale regulatory mechanisms is still lacking. This article provides a comprehensive overview of tea leaf development in terms of these four dimensions. Furthermore, key research gaps (compared with the research on model crops and other horticultural crops) are described, while future research directions in this field are discussed. This review provided herein offers a theoretical basis for improving tea varieties and developing efficient cultivation practices.

## Graphical Abstract

**Figure:**
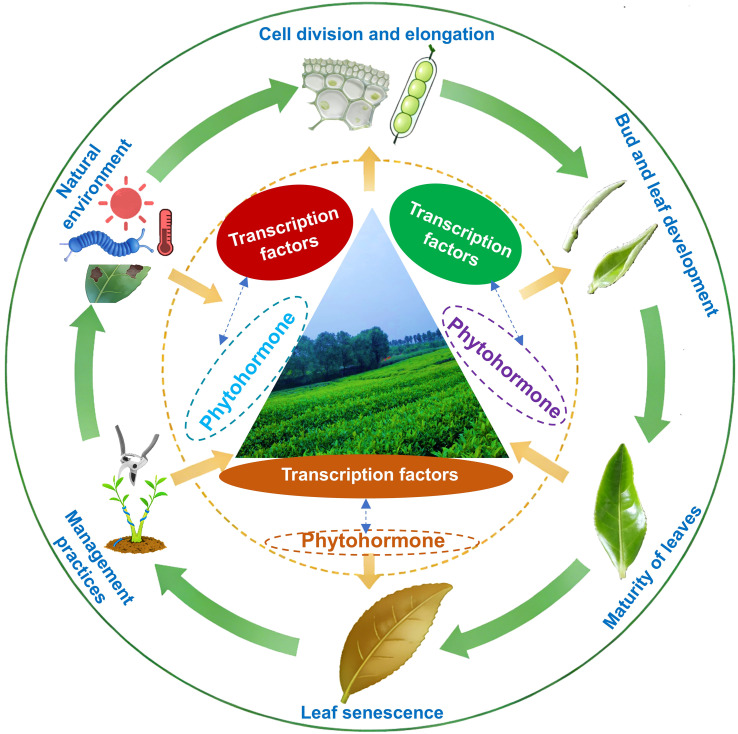

## 1 Introduction

As a globally significant economic crop with a long cultivation history, tea plants are an important resource for the production of beverages consumed worldwide, with critical contributions to economic development. Tea leaf quality and yield are determined by plant growth and foliage developmental states. Considering leaves are the core organs for photosynthesis, respiration, and metabolism, their morphological structure, developmental progression, and physiological functions directly influence the accumulation of key quality-related components, including polyphenols, caffeine, and amino acids, with implications for beverage quality and the economic value of tea leaves (Tan et al., 2023, 2024). Tea plants at different developmental stages have distinct industrial uses. For example, fresh shoots with only buds are used to produce high-end teas, such as ‘Zhuyeqing’ and ‘Baihaoyinzhen’ (Liu et al., 2025; Bao et al., 2025), whereas shoots with one bud and one leaf are the raw material for famous teas, including ‘Longjing’ and ‘Biluochun’ (Aaqil et al., 2023). By contrast, mature leaves are the core raw material for oolong tea, such as ‘Wuyi rock tea,’ and dark teas (Zhou et al., 2025; Lv et al., 2013). Thus, there is considerable theoretical and practical value to conducting thorough investigations of the regulatory mechanisms governing tea leaf development. The generated data may be relevant to optimizing cultivation practices and breeding high-yielding and high-quality tea varieties, ultimately advancing the development of the tea industry.

Tea leaf formation involves a complex biological process regulated by intrinsic genetic mechanisms, environmental factors, and cultivation practices (i.e., multi-dimensional and multi-layered regulation). During leaf morphological development, leaf primordia differentiate via cell division, cell elongation, and tissue differentiation, ultimately resulting in mature leaves with specific morphological features. This process depends on a series of critical biological activities, including cell cycle regulation, establishment of cell polarity, and organ pattern formation. Elucidating the associated regulatory mechanisms may provide a foundation for clarifying leaf developmental patterns (Zhao et al., 2025a; Wang et al., 2025; Shang et al., 2022). In terms of endogenous regulation, intrinsic regulatory factors, such as hormone signals [e.g., auxin (IAA), cytokinin (CTK), and gibberellin (GA)] and transcription factors, precisely govern each leaf developmental stage through intricate signaling networks. The identification and functional validation of key regulatory genes is crucial for elucidating the molecular mechanisms underlying leaf development (Yu et al., 2021; Shen et al., 2019). In addition, considering tea plants are perennial woody species that can grow over long periods in natural environments, their leaf development is influenced by external factors, including light, temperature, moisture, and soil nutrient availability. Moreover, during tea cultivation, artificial management practices (e.g., pruning, fertilization, irrigation, and shading) serve as crucial human interventions. These practices can regulate tea plant growth and nutrient status, thereby affecting leaf development and quality (Zhang et al., 2023a; Yuan et al., 2016; Zhou et al., 2025). Precisely regulating leaf development through scientifically informed management practices is a key research objective related to enhancing tea cultivation.

Rapid advances in modern biological techniques, including those related to molecular biology and multi-omics integrated systems biology, have recently led to significant progress in research on the regulation of tea leaf development. Several important research findings have been reported in diverse areas, including morphogenesis mechanisms, endogenous regulatory networks, environmental response mechanisms, and artificial management techniques. Nevertheless, studies conducted to date have several limitations. Notably, gene functions have not been thoroughly investigated. Nearly complete mutant libraries and functional verification systems have been established for key genes regulating leaf development, including *KNOX* and *OsLC1*, in the model plants *Arabidopsis thaliana* and *Oryza sativa* (Challa et al., 2021; Wu et al., 2025). However, only a few tea genes, such as *GRF* and *GIF* family members (Wu et al., 2017a), have been preliminarily functionally characterized. Moreover, integrated regulatory networks in tea have not been elucidated. Multi-omics studies on leaf development have been conducted for horticultural crops, such as tomato and grape (Li et al., 2024a; Fang et al., 2019), but related research on tea plants has been limited to isolated analyses of single factors, including phytohormones or transcription factors. Hence, the mechanism underlying environmental effects remains unclear. Although the cross-talk between light and temperature signals has been thoroughly elucidated in model crops (Tan et al., 2023b), the adaptive regulatory mechanisms mediating tea plant responses to specific alpine environmental conditions, such as low temperatures and high light intensity, are relatively unknown. Thus, this article provides an overview of the progress in domestic and international research on tea leaf development, with a particular focus on multi-dimensional regulation involving four core components: (1) structural dimension: conserved and species-specific tea leaf morphogenesis; (2) molecular dimension: endogenous regulatory network formed by hormones and transcription factors; (3) environmental dimension: regulatory effects of natural environmental factors on leaf development; and (4) artificial dimension: precise effects of cultivation practices on leaf development. More specifically, current findings and existing issues are summarized and future research directions are discussed. Relevant insights provided herein may be useful for further elucidating the mechanisms regulating tea leaf development, while also promoting the efficient development of a high-quality tea industry.

## 2 Fundamental tea leaf morphogenesis processes

### 2.1 Universal framework of leaf morphogenesis

**Figure 1:**
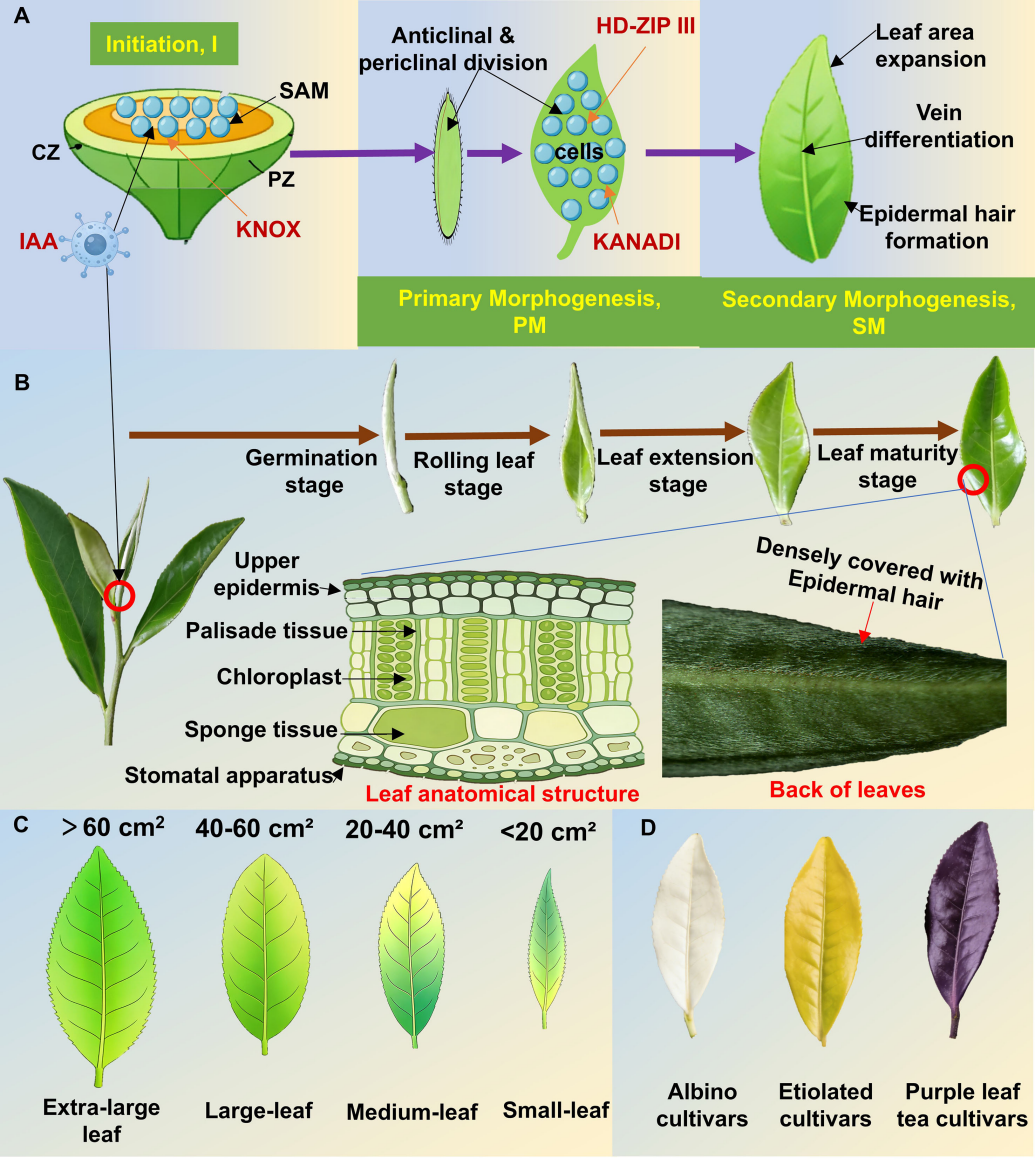
Universal framework of plant leaf morphogenesis and the specific characteristics of tea leaf morphogenesis. **(A)** The universal framework of plant leaf morphogenesis. **(B)** The specific characteristics of tea leaf morphogenesis. **(C)** The model of different leaf shape types including extra-large leaf, large-leaf, medium-leaf, and small-leaf. **(D)** The model of different leaf color types including albino cultivars, etiolated cultivars, and purple leaf tea cultivars. Auxin (IAA); Peripheral zone (PZ); Central zone (CZ); Shoot apical meristem (SAM).

Leaf morphogenesis may be represented by a chronological three-stage regulatory model, with each stage precisely controlled by gene–hormone interaction networks (**Figure 1A**). In the first stage (leaf primordium initiation phase), cells in the peripheral zone of the shoot apical meristem (SAM) differentiate into leaf primordia under conditions in which *KNOX* expression is silenced and IAA contents are increasing (Barton, 2010; Dengler and Tsukaya, 2001). In the second stage (primary morphogenesis phase), leaf primordia establish dorsal–ventral axis polarity through the mutually exclusive expression of HD-ZIP III family transcription factor genes and *KANADI* genes (Burian et al., 2022). In the third stage (expansion and secondary morphogenesis phase), the leaf surface area increases owing to cell division and elongation, while secondary structural development is completed, including vein differentiation and trichome formation (Wang et al., 2021a). SAM functions as a “signaling hub” for leaf development, comprising a central zone and a peripheral zone. Pluripotent stem cells are concentrated in the central zone and maintain the self-renewal capacity of SAM. By contrast, peripheral zone cells respond to regulatory signals to initiate leaf primordial differentiation. Changes in these two zones synergistically ensure orderly leaf development (Barton, 2010).

### 2.2 Specific tea leaf morphogenesis characteristics

Tea leaf morphogenesis represents the core process mediating leaf adaptations to climatic conditions and ensuring physiological functions are completed, with multi-dimensional specific characteristics. In terms of morphological changes, tea leaves proceed through various stages, such as germination, rolling leaf, leaf extension, and leaf maturation (**Figure 1B**). Mature leaves predominantly adopt a long-elliptical or lanceolate shape, featuring an acuminate apex with a small obtuse tip as well as undulate and crenate margins. This morphology simultaneously maximizes the photosynthetic surface area and minimizes mechanical damage associated with rainfall erosion (Xiao et al., 2024). In terms of texture, mature tea leaves are thick and leathery, with a waxy surface and a well-developed cuticle. This enhances water retention, while also protecting against pests and pathogens and forming a crucial structure for drought tolerance (Chen et al., 2020; Chen, 2021; Zhang et al., 2021a). Leaf adaptations are even more pronounced at the microscopic level (**Figure 1B**). The upper epidermis has tightly packed cells, while the lower epidermis is densely studded with stomata. This arrangement minimizes transpiration loss under intense light, while maintaining efficient gas exchange. The distinct differentiation of mesophyll tissue results in tightly arranged palisade cells rich in chloroplasts (Li et al., 2023; Nagarajah, 1979; Hideaki et al., 1976). Spongy tissue contains large intercellular spaces that facilitate the transport of photosynthetic products. This structural adaptation enables tea plants to maintain high photosynthetic efficiency, even under shaded conditions (Terashima and Saeki, 1983; Wijeratne et al., 2009; Squire, 1977).

The specificity of tea leaf developmental dynamics must be considered (**Figure 1B**). New leaves are initially yellowish-green, but they gradually become dark green as they mature. The undersides of young leaves are densely covered by fine hairs, which are rich in polyphenols and amino acids (i.e., essential compounds affecting tea quality) (Xiang et al., 2021; Cao et al., 2020). Leaf growth patterns have distinct seasonal trends: spring shoots develop rapidly with short morphological maturation cycles, while autumn shoots grow slowly and develop relatively robust structures. This diversity in morphological development represents an adaptive strategy evolved by tea plants over time, ensuring their survival, with potential implications for improving tea quality (Dai et al., 2021; Zhang et al., 2020a; Liu et al., 2017).

The differentiation of tea tree varieties has resulted in significant diversity in leaf morphology, which can be divided into four categories on the basis of mature leaf area: extra-large leaf, large-leaf, medium-leaf, and small-leaf (Compilation Committee, 2001) (**Figure 1C**). Leaf morphology is closely related to adaptability and quality. Extra-large leaf, including those of ‘Mengku daye’, exceed 60 cm^2^ and are wide and thick. Additionally, they are mostly elliptical in shape and suitable for hot and humid environments (Wang et al., 2022). They are mainly distributed in tea-growing regions along the southwestern border of China, including southern and southwestern Yunnan, and are appropriate for producing high-end Pu-erh and ‘Dianhong’ tea. Large-leaf (40–60 cm^2^), such as those of ‘Yunnan daye’, tend to be thick with an elliptical shape. Considering they are suitable for warm environments (Chen et al., 2022b; Ye et al., 2022), they are primarily distributed in southwestern China, such as Yunnan and Guangxi. In addition, they have a high substances, serving as high-quality raw materials for Pu-erh and black tea. Medium-leaf (20–40 cm^2^) produced by different tea cultivars, including ‘Qimen’ and ‘Fuding dabaicha’, have a long elliptical shape. Moreover, they are highly adaptable to cold and drought conditions, with a balanced composition (Wu et al., 2026). They are typically distributed in central and southern China, such as Fujian, Guangdong, and Hunan, where they are used to produce multiple teas, including green tea and black tea. Small-leaf (<20 cm^2^), which are produced by ‘Longjing’, ‘Biluochun’, and other cultivars, are narrow and thin, with a lanceolate shape. They are generally resistant to cold stress and are a rich source of amino acids and aroma substances (Chen et al., 2022b). Furthermore, they are mainly distributed in East China regions, such as Zhejiang, Jiangsu, and Anhui, serving as the core raw materials of famous green teas.

The diversity in leaf coloration among tea varieties is due to differences in the synthesis and accumulation of color-related compounds, including chlorophyll, carotenoids, and anthocyanins. Tea leaf colors can be divided into three main categories: albino cultivars, etiolated cultivars, and purple leaf tea cultivars (**Figure 1D**). Albino cultivars, including those of ‘Anjibaicha’, appear white before turning green. They also have a high amino acid content, making them suitable for producing high-end green tea (Li et al., 2011; Xu et al., 2017). Etiolated cultivars, which are produced by ‘Zhonghuang No.1’ as well as other cultivars, appear yellowish-green or golden during the growth period. They have a low chlorophyll content, but a high carotenoid content, and contain prominent amino acids and aroma compounds, making them a high-quality raw material for green tea production (Song et al., 2017). purple leaf tea cultivars, such as those of ‘Zijuan’, are the result of the accumulation of large amounts of anthocyanins. These leaves typically contain substantial amounts of polyphenols and antioxidants with beneficial effects on taste and human health (Lv et al., 2015).

## 3 Endogenous mechanisms regulating tea leaf development

Endogenous regulatory mechanisms have critical effects on tea leaf development, primarily relying on endogenous hormone signaling pathways and target gene expression regulated by specific transcription factors. These two processes form a “hormone–transcription factor–target gene” regulatory network.

### 3.1 Regulatory effects of endogenous hormones

#### 3.1.1 Growth-promoting hormones

**Figure 2:**
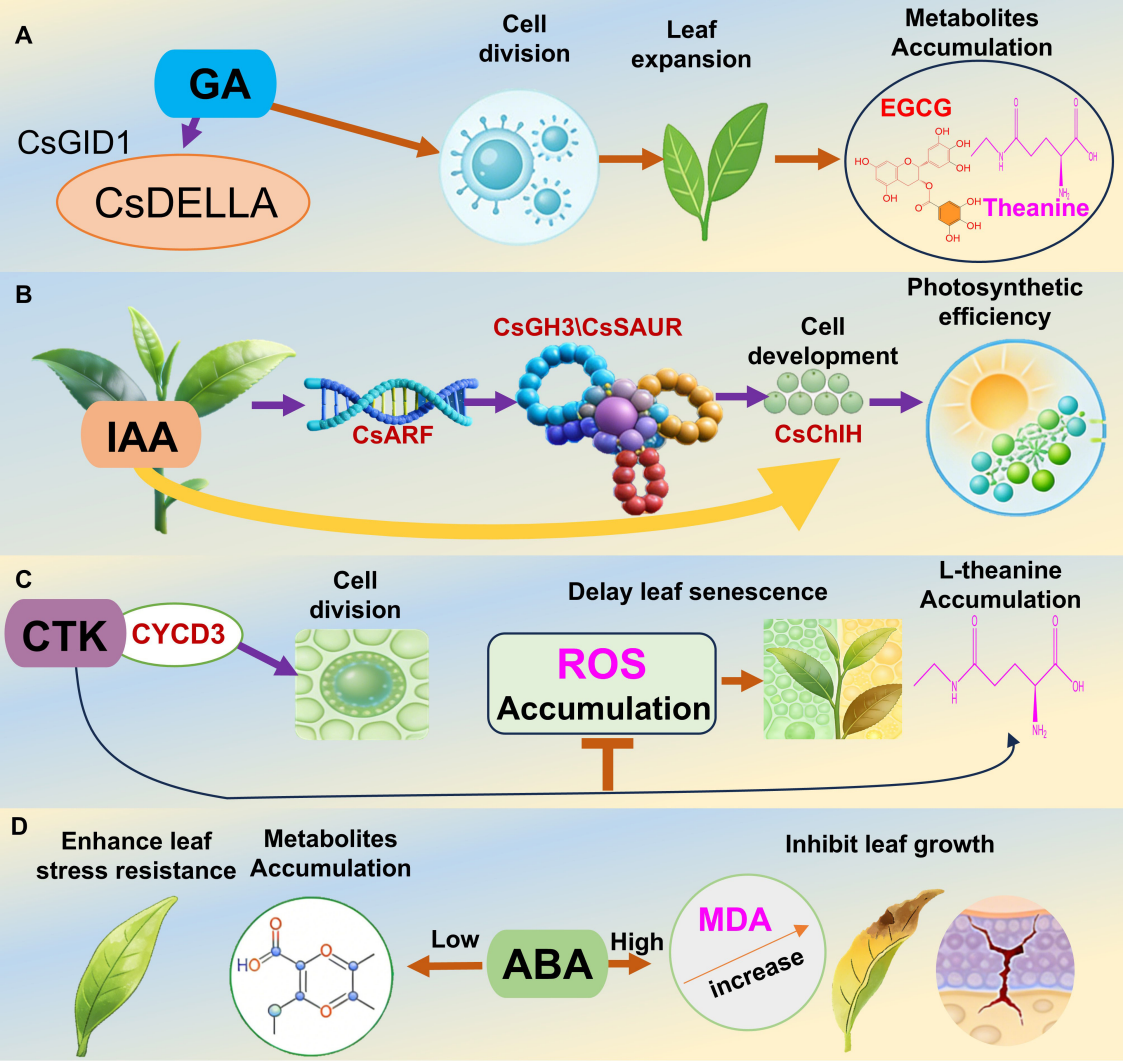
Endogenous hormones regulate the development of tea plant leaves. **(A)** Regulation of gibberellin on leaf development in tea plants. **(B)** Regulation of auxin on leaf development in tea plants. **(C)** Regulation of cytokinin on leaf development in tea plants. **(D)** Regulation of abscisic acid on leaf development in tea plants. Gibberellin (GA); Cytokinin (CTK); Auxin (IAA); Abscisic acid (ABA).

Growth-promoting hormones drive tea leaf growth by regulating cell division, elongation, and differentiation. As shown in **Figure 2A**, GA is a key plant hormone that plays a central regulatory role in tea leaf morphogenesis and physiological maturation (Yang et al., 2020). Its regulatory mechanisms span the entire leaf developmental cycle, from the initiation of leaf primordial differentiation to leaf senescence and abscission, which are precisely controlled and coordinated by multiple pathways (Li et al., 2021; Huang et al., 2026). Earlier research showed that GA can activate the expression of *CsGID1* and *CsDELLA* (core genes in the tea plant GA signaling pathway), ultimately relieving the inhibitory effect of DELLA proteins, upregulating the expression of CTK synthesis-related genes, accelerating cell proliferation and volume expansion in leaf primordia, and significantly influencing final leaf size and thickness (Li et al., 2021). At the physiological and metabolic levels, GA regulates the expression of genes encoding chlorophyll synthesis-related enzymes in tea leaves, thereby increasing the chlorophyll *a/b* ratio and photosystem II activity, while simultaneously promoting the accumulation of characteristic metabolites (e.g., flavonoids and amino acids) (Peng et al., 2013; Yang et al., 2020; Liu et al., 2020; Awon et al., 2024). GA also enhances leaf antioxidant enzyme activities, optimizes leaf developmental processes, and positively regulates tea leaf morphological and structural development, ultimately improving leaf stress resistance (Bai et al., 2025; Ye et al., 2025).

IAA is the main hormone regulating leaf development (**Figure 2B**). Its concentration gradients and polar transport are key intrinsic determinants of tea leaf morphogenesis, differentiation of physiological functions, and stress resistance (Hussain et al., 2021). At the cellular level, IAA relies on a PIN-mediated polar transport system to establish precise concentration gradients between the apical meristem and young leaves. High IAA concentrations drive the polar elongation of leaf primordial cells, while low IAA concentrations induce lateral cell division. Together, these processes regulate the spatiotemporal dynamics of cell division and elongation, which influences leaf size, thickness, and spatial configuration (Hao et al., 2019). At the molecular regulatory level, IAA activates tea-specific transcription factor families to accelerate the cell wall remodeling and cell expansion (Zhang et al., 2020b; Wang et al., 2024a; Huang, 2025). Notably, IAA and CTK have significant synergistic regulatory effects: they increase photosynthetic efficiency in tea leaves by upregulating the expression of chlorophyll synthesis-related genes, while also targeting core genes in the tea polyphenol biosynthesis pathway, ultimately influencing tea quality (Zhu et al., 2023; Ye et al., 2024a). Furthermore, IAA may play a central regulatory role in pruning-induced leaf bud emergence and growth (Zhang et al., 2023b). The metabolic homeostasis of IAA leads to the differential expression of related functional genes and the modulation of protein interaction networks that precisely regulate tea plant growth and development (Wang et al., 2020a).

CTK, which is one of the main hormones regulating plant cell proliferation and senescence, has critical regulatory effects on the entire life cycle of tea leaves, from leaf primordial differentiation and morphogenesis to functional maturation (**Figure 2C**). According to published research, CTK can regulate cell cycle-related gene expression to accelerate cell division and provide the cellular foundation for rapid leaf expansion and morphogenesis (Thirugnanasambantham et al., 2020). In addition, CTK has dual regulatory effects on leaf functions. Specifically, it significantly delays chlorophyll degradation, maintains photosynthetic membrane stability, and enhances photosynthetic rates (Kakkar and Nagar, 1996), while also modulating theanine synthesis and promoting the directional accumulation of characteristic tea plant components, thereby influencing tea quality (Peng et al., 2013). Furthermore, CTK enhances leaf antioxidant system activity, restricts reactive oxygen species (ROS) accumulation, and delays leaf senescence (Zhao et al., 2025b).

#### 3.1.2 Abscisic acid: dual regulatory roles in leaf development

Abscisic acid (ABA) has dual regulatory effects on tea leaf development (**Figure 2D**). Low ABA concentrations significantly increase leaf soluble solids, proline contents, and antioxidant enzyme levels, leading to blade structural stability. ABA also maintains photosynthetic efficiency under cold conditions by regulating drought stress-related gene expression and altering metabolite levels (Gai et al., 2020), mitigating damages to tea leaves from adverse conditions (especially cold and drought) (Li et al., 2019) and ensuring normal leaf development (Wang et al., 2024b). However, high ABA concentrations increase malondialdehyde (MDA) levels, cell membrane permeability, and damages to tea seedlings, with detrimental effects on leaf growth (Zhou et al., 2020).

### 3.2 Regulatory networks involving transcription factors

**Figure 3:**
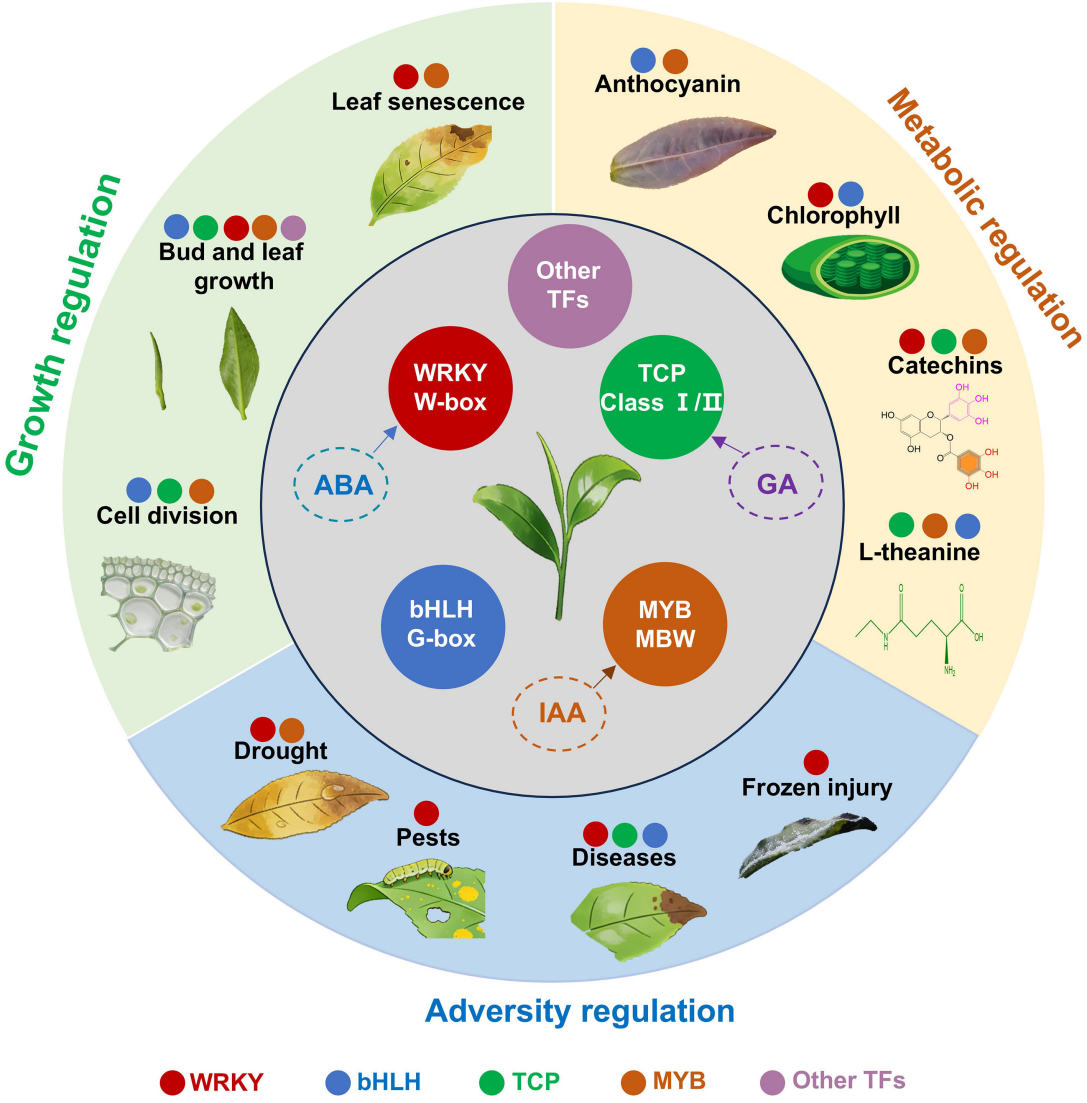
Regulatory networks of transcription factors in tea leaf development. Transcription factors (TFs); Gibberellin (GA); Abscisic acid (ABA); Auxin (IAA).

Tea leaf development is a complex process regulated by multiple genes. Transcription factors influence leaf morphological development by spatiotemporally controlling relevant gene expression. They have been linked to secondary metabolic pathways that affect the accumulation of quality-related components. In recent years, transcription factors, such as WRKY, bHLH, TCP, and MYB family members, have been widely reported to regulate tea leaf development (**Figure 3**). The primary functions of core transcription factor families are described below.

#### 3.2.1 WRKY family

WRKY family members are plant-specific transcription factors that regulate target gene expression by binding to the W-box promoter element. They play a central role in the multi-dimensional regulation of tea leaf development. Spatiotemporal WRKY expression patterns during leaf development suggest the encoded transcription factors regulate leaf growth and development (Wang et al., 2019). During leaf morphogenesis, *CsWRKY51* overexpression modulates the tea leaf structure (e.g., leaf curling) and chlorophyll accumulation (Chen et al., 2025a). *CsWRKY57* expression is correlated with leaf senescence, with high expression levels in senescent leaves suggestive of regulatory effects on senescence (Guo et al., 2017a). Meanwhile, some WRKY transcription factors can help maintain leaf structures and functions under adverse conditions. For example, CsWRKY26 and CsWRKY48 maintain normal leaf cell division and extension under drought conditions, thereby ensuring that leaf morphogenesis proceeds normally (Chen et al., 2021a). CsWRKY17 can respond to herbivorous insect infestations to maintain the integrity of young leaf tissue and stabilize leaf development-related processes (Liu et al., 2021a). Both CsWRKY2 and CsWRKY21 regulate the effects of low temperatures on leaf cell division and differentiation to facilitate normal germination and growth of young leaves (Wang et al., 2016; Mi et al., 2024; Wang et al., 2019). CsWRKY3 can respond to tea geometrid feeding to stabilize leaf photosynthetic functions and maintain leaf growth and development (Ge et al., 2018). By contrast, CsWRKY14 is responsive to anthracnose and tea leaf spot pathogens; it maintains leaf structures and functions, while ensuring leaves develop normally throughout the growth period (Liu et al., 2021b). In addition, WRKY transcription factors can coordinate cell growth and metabolic homeostasis, while mediating normal leaf development and functions. Specifically, *CsWRKY12* is preferentially expressed in young leaves and participates in the regulation of substance accumulation and tissue maturation during leaf development, with positive effects on leaf functions (Zhang et al., 2024a).

#### 3.2.2 bHLH family

Members of the bHLH transcription factor family affect tea leaf developmental processes and metabolic networks by binding directly to cis-acting elements in target gene promoter regions or by interacting with other transcription factors to form regulatory complexes. In terms of morphogenesis, *CsbHLH2* is expressed in tea buds, young leaves, and mature leaves, with peak expression levels in young leaves. The encoded protein primarily regulates cell division and proliferation, thereby contributing to early leaf development and morphogenesis (Han et al., 2015). CsPIF3b activates *CsHEMA* and *CsPOR* expression to help regulate chloroplast development (Zhang et al., 2021b). CsbHLH024 and CsbHLH133 interact with CsTTG1, which regulates tea trichome formation, thereby promoting late trichome development (Liu et al., 2021c). Furthermore, certain *CsbHLH* family genes encode transcription factors that indirectly regulate leaf development by controlling metabolic homeostasis, integrating hormone signals, and enhancing tea plant stress resistance (Han et al., 2025; Liu et al., 2021c; Tian et al., 2025). For example, CsbHLH89 targets important genes in the anthocyanin synthesis pathway (*CsANS* and *CsDFR*). By promoting anthocyanin biosynthesis and accumulation, it influences leaf coloration, while also increasing antioxidant activities and regulating leaf development (Zhang et al., 2024b). CsbHLH35 interacts with the MYB family transcription factor CsMYB213 to form a heterodimeric complex that activates the expression of the key theanine synthesis-related gene *CsTSI*. This complex also responds to nitrogen signals to maintain leaf nitrogen metabolic equilibrium, which is important for normal leaf growth and development (Xie et al., 2025). CsbHLH62 enhances the positive effects of CsNAC17 on *CsRPM1* expression, leading to improved resistance to anthracnose (Han et al., 2025).

#### 3.2.3 TCP family

The TCP transcription factor family is divided into two subfamilies: Class I and Class II. Class II, which is further subdivided into the CIN branch and the CYN/TB1 branch, is critical for the regulation of tea leaf morphogenesis and plant growth (Wu et al., 2017b). In terms of tea plant morphogenesis and quality stability, CsTCPs directly regulate SAM development and leaf cell division, thereby affecting leaf morphology, physiological functions, and quality (Yu et al., 2021). CsTCP2, which belongs to the CIN branch of Class II, is associated with miR319c. These two elements synergistically regulate apical bud emergence to fine-tune tea plant growth and development (Liu et al., 2019). CsTCP2 regulates photosynthetic pathways and various free amino acid synthesis pathways. By influencing photosynthesis, it modulates theanine synthesis to positively affect tea leaf quality (Mao et al., 2025). Furthermore, TCP also maintain the integrity of leaf function under adverse conditions. For example, CsTCP10 decreases disease-related leaf damage by activating the expression of disease resistance genes (Liu et al., 2022). *CsTCP11* is co-expressed with *CsCHAT1* to protect tea leaves from herbivorous pests (Gu et al., 2025).

#### 3.2.4 MYB family

In terms of leaf growth and morphogenesis, CsMYB6 activates the cell cycle gene *CsCDKB1*. The resulting division of apical meristematic cells and tender stem elongation influence the leaf emergence rate (Li et al., 2022a). The phosphorylation of CsMYB4a disrupts its ability to inhibit the expression of phenylpropanoid pathway genes. This is associated with the activated expression of the transcription factor gene *YABBY5*, which helps to maintain leaf polarity (Ma et al., 2025). Artificial selection-induced single nucleotide polymorphisms in *CsMYB1* can increase trichome density and improve insect resistance (Li et al., 2022b), revealing the role of *CsMYB1* during tea domestication. Several MYB transcription factors mediate normal leaf development and functional establishment by regulating secondary metabolism. CsMYB2/CsMYB26 promote catechin accumulation by activating the expression of flavonoid synthesis-related genes, such as CsF3′H, leading to increased tea leaf antioxidant activities (Wang et al., 2018a). CsMYB113 binds to the promoter of the anthocyanin synthesis-related gene *CsANS* to modulate expression and significantly increase anthocyanin contents, leading to the formation of purple buds (Shui et al., 2023). By contrast, CsMYB73 regulates nitrogen allocation by suppressing the expression of the theanine synthase gene *CsTSI* (Wen et al., 2020). Of the MYB transcription factors that facilitate normal leaf functions under adverse conditions, CsMYB1/CsMYB2 upregulate the expression of the antioxidant enzyme-encoding gene *CsSOD* to limit leaf oxidative damage (Li et al., 2022a). Studies also indicate that MYB transcription factor functions may depend on the formation of the MBW (MYB–bHLH–WD40) complex (e.g., CsMYB8 interacting with bHLH and WD40 domains) and tissue-specific gene expression (Hu et al., 2024a).

#### 3.2.5 Common regulatory patterns and cross-family interactions of transcription factors

The four transcription factor families described in previous sections do not regulate leaf development independently. Instead, they form a complex synergistic regulatory network with common regulatory patterns. First, all four families are involved in core leaf development-related processes, including cell cycle regulation, polarity establishment, tissue differentiation, and senescence, but their functions differ. TCP and MYB family members are the core regulators of early leaf morphogenesis and cell division (Yu et al., 2021; Li et al., 2022a). By contrast, WRKY and bHLH transcription factors have important regulatory effects on mature leaf functions and secondary metabolism (Guo et al., 2017a; Liu et al., 2021c). Second, transcription factor families form a hierarchical regulatory network through direct interactions. For example, MYB and bHLH transcription factors form an MBW complex that serves as the core regulator of anthocyanin and proanthocyanidin biosynthesis in leaves (Hu et al., 2024a). Interestingly, bHLH transcription factors can interact with WRKY transcription factors to co-regulate disease resistance-related responses as well as leaf development (Xie et al., 2026). TCP transcription factors can regulate the expression of genes encoding MYB transcription factors, while linking leaf morphogenesis to secondary metabolism (Yu et al., 2021). Third, all four transcription factor families are important for linking hormone signals to leaf development (**Figure 3**). TCP transcription factors respond to GA signals to regulate cell elongation and leaf expansion (Wu et al., 2017b). MYB transcription factors are regulated by IAA to control leaf polar growth (Li et al., 2022a). WRKY transcription factors, which are key components of the ABA signaling pathway, can link stress responses to leaf development (Samarina et al., 2019). This hormone–transcription factor regulatory network ensures that tea leaves develop in an orderly manner during different growth stages and under various environmental conditions.

### 3.3 Other transcription factors and synergistic regulatory mechanisms

Many other transcription factor families also regulate tea leaf development. Genes encoding SBP-box family members are more highly expressed in bud and leaf tissues than in other tissues, reflecting potential regulatory effects on bud and leaf development (Wang et al., 2018b). Tea plant *GRAS* family genes, which encode transcription factors with crucial roles, have tissue- and developmental stage-specific expression patterns. These transcription factors regulate plant growth and stress responses via multiple pathways (Wang et al., 2018c). Furthermore, plant-specific lateral organ boundary domain transcription factor family genes are differentially expressed among tea plant tissues, including roots, stems, leaves, and flowers, with important roles in leaf development (Teng et al., 2018). Notably, transcription factors do not regulate leaf development independently. Instead, they frequently form synergistic regulatory networks involving hormone signaling pathways. For example, the expression of some ABA-inducible transcription factor genes, such as CsWRKY57, can activate the expression of genes associated with stress responses and metabolism. TCP family members promote cell elongation and apical bud emergence in response to GA signals, while MYB transcription factors regulated by IAA can influence leaf polar growth and secondary metabolic processes. These synergistic interactions among transcription factors and hormones facilitate the orderly development of tea leaves across different growth stages and environmental conditions.

## 4 Natural environmental regulation of tea leaf development

**Figure 4:**
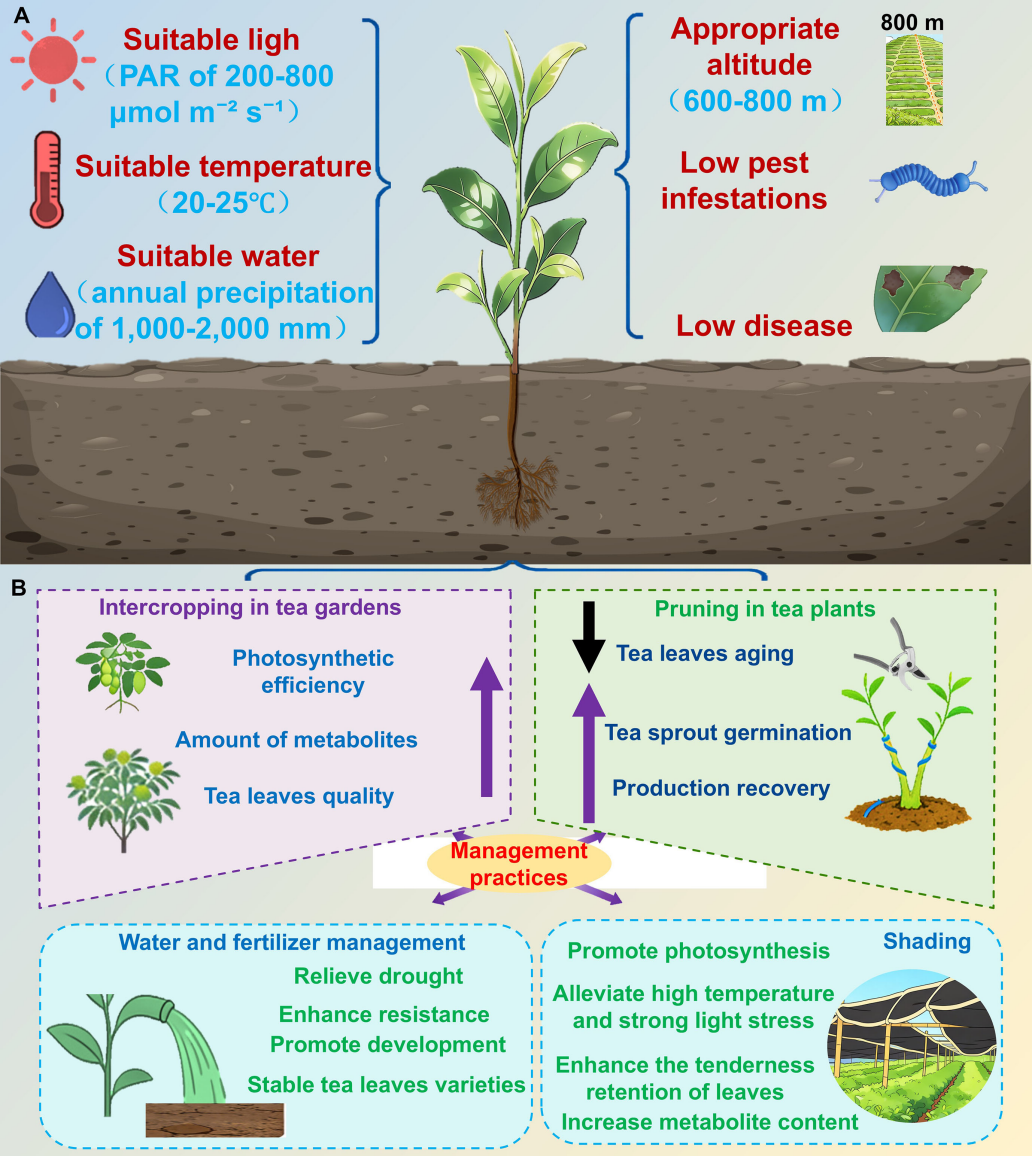
Natural environmental and artificial management regulation of tea leaf development. **(A)** Natural environmental regulation of tea leaf development. **(B)** Artificial management regulation of tea leaf development.

Natural environmental conditions are the core external inducers of tea leaf development, controlling growth and development by influencing leaf morphology, physiological functions, and metabolite accumulation. Key environmental factors include light, temperature, moisture, altitude, and biotic interactions (pests and pathogens) (**Figure 4A**). As a typical subtropical shade-tolerant C3 crop, tea is suitable for environments with a photosynthetically active radiation (PAR) of 200–800 µmol m^−2^ s^−1^ (De Costa et al., 2007), temperatures between 20 and 25 °C, annual precipitation of 1,000–2,000 mm, and relative humidity of 80%–90% (Zhang et al., 2025). These environmental conditions mainly influence leaf development through their effects on the endogenous hormone–transcription factor regulatory network described in Section 3.

### 4.1 Light

Light is an energy source for tea plant photosynthesis and a crucial signal regulating leaf development, with both light intensity and quality affecting tea plants.

#### 4.1.1 Light intensity

Moderate light intensity (PAR of 200–800 μmol m^−2^ s^−1^) positively modulates tea leaf development (De Costa et al., 2007). Morphologically, it increases the number of leaves as well as leaf area (Adeosun et al., 2022), while also intensifying leaf coloration (Manikharda et al., 2023). Physiologically, it markedly increases carotenoid and total chlorophyll contents as well as chlorophyll *a*/*b* ratios (Chen et al., 2022a), thereby increasing the light-harvesting capacity. In terms of stress tolerance, moderate light intensity during cold winters can increase net photosynthetic rates (Han et al., 2023), decrease ROS and MDA levels, mitigate membrane lipid peroxidation, and alleviate cold-induced photoinhibition (Zaman et al., 2022). A classic example is the ‘Huangjinya’ tea plant, whose leaves turn yellow because of inhibited chlorophyll synthesis under intense light (Li et al., 2016). Shading leads to increases in chlorophyll contents and leaf greening. This is mainly mediated by the light receptor CsHY5, which can regulate the expression of chlorophyll synthesis-related genes in response to changes in light intensity (Tian et al., 2017).

#### 4.1.2 Light quality

The effects of different light qualities have distinct effects on leaf development: red light promotes chlorophyll accumulation, significantly increases net photosynthetic rates (Hu et al., 2025), and stimulates bud growth (Wei et al., 2018) blue light increases carotenoid synthesis (Tian et al., 2021), promotes co-expression of photosynthesis and lipid metabolism-related genes (Wang et al., 2020b), and enhances anthocyanin and catechin accumulation (Zheng et al., 2019). UV-B light is the main inducer of anthocyanin synthesis in tea plants, efficiently degrading the CsHY5 repressor protein via the UVR8–COP1–HY5 signaling axis to activate anthocyanin synthesis-related gene expression and increase leaf anthocyanin contents (Liu et al., 2023a). At the endogenous regulatory level, different light qualities activate specific photoreceptors, regulate the expression of genes encoding bHLH and MYB transcription factors, and further modulate hormone synthesis and leaf development (Zheng et al., 2019).

#### 4.1.3 Photoperiod

Photoperiod is a key environmental factor that regulates tea leaf growth, development, and quality. At the phenotypic and physiological levels, long-day conditions significantly enhance bud and leaf germination, leaf extension, chlorophyll synthesis, and photosynthetic efficiency, while maintaining nutrient-related growth advantages (Hu et al., 2024b). For example, ‘HuangKui’ growth is optimized if plants at the one bud and one leaf stage are cultivated using a 16-h light/8-h dark cycle (Hu et al., 2024b). In addition, the expression levels of key genes, including *CHLE*, *CHLP*, *CLH*, *POR*, and *P450*, which encode enzymes mediating chlorophyll synthesis, reportedly increase in response to an exposure to continuous light (Hu et al., 2024c). At the molecular level, photoperiod-related signals are perceived and transduced through core biological clock pathway components, including *CsLUX* and *CsELF*, which regulate rhythmic changes in photosynthetic parameters and stomatal opening, thereby modulating tea leaf physiological activities (Sun et al., 2025; Hu et al., 2024d). Furthermore, recent research revealed that the photoperiod along with light quality and temperature can fine-tune leaf development and quality-related metabolism via epigenetic modifications such as histone methylation (e.g., CsSDG36-mediated H3K4 methylation) (Wang et al., 2025).

### 4.2 Temperature

Temperature regulates tea leaf development through its effects on enzyme activities, hormone metabolism, and photosynthesis. The optimal range for these processes is 20-25 °C (Qiu et al., 2024a). Temperature stress mainly affects leaf development by disrupting endogenous hormone homeostasis and regulating the expression of genes encoding stress-related transcription factors. Low temperatures (<10 °C) significantly inhibit leaf development. More specifically, they decrease the accumulation of growth-promoting hormones, such as GA and IAA, causing a substantial decrease in bud growth rates (Wang et al., 2023). Morphologically, young tea leaves stop growing and become brown and wilted, with frost damage-associated spots in severe cases (Chen et al., 2024). Physiologically, chlorophyll and carotenoid contents markedly decrease (Chen et al., 2024), impairing photosystem II functions and reducing Fv/Fm values (maximum photochemical efficiency of photosystem II) to a notable extent. These changes adversely affect light energy conversion efficiency, thereby decreasing photosynthetic rates (Oh and Koh, 2014). At the molecular level, low temperatures can activate the expression of genes encoding CBF/DREB transcription factors and cold-responsive WRKY transcription factors, including CsWRKY2 and CsWRKY21, while also regulating the expression of stress response-related genes and maintaining basic leaf physiological functions (Wang et al., 2016; Mi et al., 2024; Wang et al., 2019). High temperatures (35–40 °C) similarly impede leaf development. In terms of their effects on photosynthesis, high temperatures substantially decrease maximum net photosynthetic rates, exacerbate photosystem II photoinhibition, and inhibit light use and conversion by the photosynthetic system (Li et al., 2015). Moreover, increased temperatures alter hormone synthesis and metabolism in tea plants, leading to decreases in IAA, GA3 (Tang et al., 2023), and ABA contents (Liu, 2020), eventually disrupting hormonal equilibrium and inhibiting growth. At the cellular level, high temperatures increase leaf cell membrane permeability, MDA contents, and relative membrane conductance, thereby exacerbating membrane damage and impairing substance transport (Seth et al., 2021). Notably, a previous study determined that each day of extreme heat (34 °C < T_mean_ ≤ 36 °C) can decrease yields by approximately 3.7% (Yan et al., 2021). At extremely high temperatures exceeding 48 °C, heat-damaged leaves cannot recover, potentially leading to total yield losses (Jiang et al., 2014). At the molecular level, high temperatures can activate the expression of genes encoding heat shock proteins and heat stress-related transcription factors that regulate the antioxidant system to limit heat-induced leaf damage (Tan et al., 2024b).

### 4.3 Water and altitude

#### 4.3.1 Water

Water stress (drought or waterlogging) inhibits tea plant growth. Drought stress is a key factor constraining tea leaf development, with mild drought stress alone causing morphological changes, including decreased leaf areas and increased leaf color intensity. Physiologically, mild drought induces stomatal closure, resulting in suppressed photosynthesis and respiration (Qian et al., 2018), while also promoting lignin accumulation to enhance cell wall stability and minimize water loss (Gu et al., 2020). An exposure to severe drought can lead to leaf wilting or even death (Guo et al., 2017b). Metabolically, it decreases leaf amino acid and organic acid contents, with significant detrimental effects on tea quality (Malyukova et al., 2022). At the molecular level, drought stress activates the ABA signaling pathway (Gai et al., 2020), upregulates the expression of genes encoding drought-responsive WRKY transcription factors, regulates stomatal movement and stress response-related gene expression, and maintains basic leaf development (Chen et al., 2021a). Notably, tea plants that undergo a drought-conditioning treatment are better able to adapt to a subsequent exposure to short-term drought stress than untreated plants (e.g., increased antioxidant enzyme activities and decreased ROS accumulation) (Chen, 2022), offering insights for optimizing tea garden management practices in arid regions. Conversely, waterlogging induces root hypoxia and decay, thereby impeding nutrient uptake and preventing new shoot growth, with plant death a possibility under severe conditions (Cisse et al., 2023).

#### 4.3.2 Altitude

Altitudinal changes indirectly influence tea leaf development because of the associated differences in environmental factors, including temperature, light exposure, and humidity. The optimal altitude for tea cultivation is generally between 600 and 800 m. Studies on tea gardens distributed along the altitudinal gradient of Mount Lu revealed that as the altitude increased, the tea leaf transpiration rate, stomatal conductance, intercellular CO_2_ concentration, and photosystem II photoinhibition decreased, which was in contrast to increases in the net photosynthetic rate and water use efficiency (Cheng et al., 2019). In terms of leaf morphology, increases in altitude lead to several adaptive changes. More specifically, tea leaves thicken. This is accompanied by increases in the number of palisade tissue layers, palisade tissue:sponge tissue ratio, specific leaf weight, and epidermal hair density. However, the area of individual leaves decreases. These morphological changes in response to high-altitude environmental conditions positively affect tea leaf photosynthetic activities and stress resistance (Wang and Cheng, 2021b). In addition, high-altitude environments can induce the production of metabolites conducive to leaf morphological development and quality formation. For example, high altitudes increase free amino acid and fatty acid contents in leaves, resulting in the accumulation of flavonoids and soluble proteins (Tian et al., 2024). Although catechin synthesis is inhibited under high-altitude conditions (Tian et al., 2024), the increase in flavonoid compound contents compensates for this deficiency, helping to maintain high total phenolic contents and antioxidant activities (Fibrianto et al., 2024), ultimately resulting in refreshing tea flavors. A typical case is tea produced in the mountainous region (approximately 1,000 m from the seaside) in Taiwan. Leaves from tea plants cultivated in this region have a high amino acid content, low phenol:ammonia ratio, and unique floral and fruity aromas, making them a typical representative of high-quality tea leaves from mountainous regions (Cai et al., 2014).

### 4.4 Pests and diseases

Pest infestations and pathogen infections typically negatively affect tea leaf development through morphological damages and disrupted metabolic activities. Diseases primarily caused by fungal and bacterial pathogens are often major inhibitors of normal tea leaf development. Fungal diseases are the most prevalent diseases among tea plants, with anthracnose caused by different pathogens, such as *Rhizoctonia solani*, resulting in irregular lesions that disrupt photosynthetic structures (Jeyaraj et al., 2019). Bud blight caused by *Fusarium oxysporum* decreases the abundance of the quality-related metabolite epigallocatechin gallate (Chen et al., 2025b), whereas gray leaf spot and blister blight impair leaf development by damaging leaf structures (Chen et al., 2021b; Liu et al., 2021b). Symptoms of bacterial white leaf blight, which is due to *Xanthomonas oryzae pv. oryzae*, include yellowish-white streaks on desiccated leaves (Liu et al., 2021b). These diseases are common under specific environmental and cultivation conditions (e.g., high temperatures and humidity, poor ventilation, excessive nitrogen application, or unhealed pruning wounds). Notably, unclear synergistic mechanisms underlying responses to complex diseases, unknown differences in disease resistance-related gene expression between cultivars, and a lack of molecular mechanisms for effective control measures during cultivation should be addressed in future studies. Additional research is required to support the breeding of disease-resistant cultivars and optimize green control technologies. At the molecular level, pathogen infections activate jasmonic acid and salicylic acid signaling pathways, upregulate the expression of genes encoding disease resistance-related transcription factors (WRKY, bHLH, and TCP), induce the expression of disease response-related genes, and maintain leaf structures and functions (Liu et al., 2021b; Han et al., 2025; Zhou et al., 2024).

Insect infestations have multi-faceted effects on foliage. Morphologically, feeding by tea green leafhoppers can decrease the leaf area as well as internode length and diameter (Gu et al., 2024). In addition, feeding by tea looper larvae leads to extensive leaf degradation, leaving only veins in severe cases, resulting in substantial yield losses (Bhattacharyya et al., 2024). Insect pests that use their piercing–sucking mouthparts to consume leaf sap can cause bud and leaf yellowing, curling, and withering as well as inhibited growth. Their oviposition in tender shoot tissues further impedes bud and leaf elongation (Wu, 2023). Metabolically, a black-spined whitefly infestation decreases leaf polyphenol contents (Li et al., 2020), while green leafhopper feeding decreases the accumulation of key amino acids (e.g., theanine), while increasing flavonoid contents, the associated imbalance in leaf metabolism can decrease tea quality (Zhao et al., 2020). At the molecular level, insect infestations activate the jasmonic acid signaling pathway, upregulate the expression of genes encoding defense-related transcription factors, and induce the synthesis of defensive secondary metabolites and volatiles, ultimately enhancing tea tree resistance to herbivorous insects (Ge et al., 2018). Notably, moderate pest infestations may positively affect certain tea varieties. For example, moderate feeding by leafhoppers can trigger ‘Dongfang meiren’ tea tree defense responses, optimize leaf chemical compositions via the synthesis of large amounts of monoterpenes and sesquiterpenes, and alter appearance-related traits. Hence, this moderate feeding endows ‘Dongfang meiren’ tea with core qualities that distinguish it from other types of tea (Yan et al., 2025). Accordingly, pest infestations can positively and negatively alter tea leaf development and quality formation.

## 5 Artificial management of tea leaf development: cultivation-based regulation

Artificial management based on intrinsic mechanisms mediating tea leaf development and environmental response patterns involves cultivation control measures to optimize leaf growth and quality. Specific methods include intercropping, pruning, fertilization, irrigation, shading, and other agronomic practices, which are conducive to the production of high-quality and high-yielding plants in tea gardens (**Figure 4B**).

### 5.1 Intercropping

Intercropping systems in tea gardens indirectly regulate leaf development by optimizing microclimatic conditions and resource allocation, reflecting their potential utility for improving tea plant cultivation. In terms of its morphological and physiological effects, tea–chestnut intercropping can improve the distribution of light in tea gardens, leading to leaf elongation and increased leaf fresh weight (Wu et al., 2021). Additionally, tea–soybean intercropping increases soil nitrogen contents via legume-based nitrogen fixation, which promotes leaf chlorophyll a/b accumulation, while also enhancing photosynthetic electron transport efficiency, ultimately increasing the net photosynthetic rate (Li et al., 2024b). Changes in metabolism and quality due to intercropping with green manure are related to increased soil organic matter contents (Fu et al., 2018), which positively affects the accumulation of amino acids (particularly L-theanine) and soluble sugars in tea leaves (Duan et al., 2019). Intercropping tea with oyster mushrooms regulates the carbon–nitrogen balance in tea gardens via microbial metabolism, resulting in increases in the total leaf catechin content. Notably, epigallocatechin gallate levels increase significantly to substantially enhance tea antioxidant properties (Yang et al., 2024).

### 5.2 Pruning

Pruning regulates leaf emergence rhythms and senescence processes by altering apical dominance and nutrient allocation in tea plants. Hence, it is a critical practice related to tea garden canopy management. Pruning measures, which are categorized according to regulatory objectives and intensity, include light pruning (spring/autumn pruning) and heavy pruning (rejuvenation pruning). These practices are applied in distinct tea plant growth phases to ensure leaf-related processes are precisely regulated. Light pruning involves removing 3–5 cm of tender shoots from the canopy surface, which decreases the IAA concentration at lateral bud sites to release apical dominance inhibition. Pruning also stimulates CTK synthesis and transport, with the subsequent increase in the CTK-to-IAA ratio further promoting lateral bud emergence. Decreasing the ABA concentration and delaying aging-related gene expression can extend the period in which leaves are functional and the tea harvesting cycle (Ye et al., 2024b). For relatively old tea plants or those severely affected by pests and diseases, heavy pruning is required. More specifically, removing one-third to one-half of the branches from the canopy forces the tea plant to redistribute nutrients to basal buds, thereby promoting the emergence of vigorously growing new shoots (De Costa et al., 2009). These renewed shoots have relatively high chlorophyll contents and photosystem II-related gene expression levels (e.g., genes *PSBO* and *PSBA*), leading to significantly increased net photosynthetic rates in new leaves. These changes lead to canopy renewal and yield recovery (Ahmad et al., 2018).

### 5.3 Fertilizer regulation

Fertilization, which optimizes leaf physiological activities and secondary metabolism, is a critical agronomic practice for regulating tea yield and quality (Xiao et al., 2018). Nitrogen, phosphorus, and potassium fertilizers are the core agronomic inputs for regulating tea leaf development. Notably, nitrogen fertilizers have the most favorable effects on tea plant growth, quality, and yield. Nitrogen is the core raw material for theanine synthesis. Appropriate nitrogen fertilizer applications can increase the expression of the theanine synthase gene *CsTSI*, resulting in the accumulation of theanine in leaves (Xie et al., 2025). For the production of high-quality mature leaves in tea plantations, nitrogen fertilizers should be applied in the following two key periods: after the end of the tea harvest season (late September to November) and in the early stage before spring tea harvest. According to a recent study, the annual recommended application rate for nitrogen fertilizers is 200–350 kg N/hm^2^ (Qiu et al., 2024b), which is critical for maintaining high tea yields, nitrogen accumulation, and nitrogen use efficiency. Phosphorus and potassium fertilizer applications must also be carefully controlled. Phosphorus fertilizers (e.g., superphosphate) promote ATP synthesis in leaves, enhance the activity of photosynthetic enzymes, such as ribulose-1,5-bisphosphate carboxylase/oxygenase, and increase net photosynthetic rates (Khan et al., 2023). For the combined application with an organic fertilizer, a phosphorus fertilizer should be applied as a basal fertilizer after the end of the tea harvest season (late September to November), with an optimal annual application rate of 45 kg P/hm^2^ for pure phosphorus (Wang et al., 2024c). By contrast, potassium fertilizers (e.g., potassium chloride) enhance leaf stomatal regulation, improve water use efficiency, and decrease leaf wilting rates. They also influence the synthesis and proportions of flavor-related compounds, such as polyphenols and amino acids, which affect tea quality (Huang et al., 2022). A potassium fertilizer should also be applied as a basal fertilizer (124 kg/hm^2^ KCl or 156 kg/hm^2^ K_2_SO_4_) after the end of the tea harvest season (late September to November), which can significantly improve tea quality and yield (Huang et al., 2022).

Notably, both excessive and insufficient fertilization impede normal tea plant growth, development, and quality formation. For example, excessive nitrogen inputs cause excessive vegetative growth and uneven bud development, with detrimental effects on tea quality (Qiu et al., 2024b). Similarly, the over-application of phosphorus and potassium fertilizers predominantly suppresses the biosynthesis of key quality-related metabolites in tea leaves, namely flavonoids and free amino acids (Wang et al., 2024c; Huang et al., 2022). By contrast, insufficient fertilization generally restricts normal tea leaf development by triggering leaf chlorosis, decreasing photosynthetic efficiency, and adversely altering other leaf characteristics, ultimately leading to decreases in tea yield and quality (Qiu et al., 2024b). Furthermore, the ratio of nitrogen, phosphorus, and potassium in fertilizers must be considered. The optimal nitrogen:potassium ratio is 1:0.83 or 1:0.62 when potassium is derived from KCl, whereas it is 1:0.21 or 1:0.42 when potassium is provided as K_2_SO_4_ (Huang et al., 2022).

Micronutrients are indispensable for regulating tea plant growth, development, and quality formation because they are involved in key physiological processes, including photosynthesis, respiration, nitrogen metabolism, hormone synthesis, and secondary metabolism. Critical micronutrients, such as iron, manganese, zinc, and copper, contribute to chlorophyll synthesis and enzyme-catalyzed reactions, thereby ensuring leaves grow and accumulate specific compounds normally (Liu et al., 2010). In addition, boron affects the cell wall structure and reproductive development, while also improving stress resistance (Mukhopadhyay et al., 2013). Moreover, molybdenum and chlorine are involved in nitrogen use and water transport (Liu et al., 2010; Li, 2014). Appropriate amounts of selenium and germanium in tea plants can regulate physiological metabolism and increase the contents of health-promoting components in leaves (Yang et al., 2026; Su et al., 2024). Although the demand for these elements is extremely low, their deficiency or imbalance will lead to leaf chlorosis, growth retardation, and metabolic disorder. The rational application of micronutrients can promote bud and leaf growth and modulate tea plant growth vigor, which can improve the aroma, taste, and nutritional quality of the final tea product.

Moreover, as a key regulatory component of green cultivation practices, organic fertilizers primarily improve soil conditions and have long-term benefits. For example, their application enhances rhizosphere soil conditions, increases soil fertility, and improves soil structural characteristics, with positive effects on root development and subsequent leaf growth. Organic fertilizers also increase leaf chlorophyll contents to enhance photosynthesis, improve leaf stress resistance, prolong functional periods, and delay senescence. This extends the harvesting window for premium tea leaves and results in superior raw materials. Moreover, these fertilizers can significantly increase total free amino acid contents to improve tea quality, while decreasing environmental pollution due to the application of chemical fertilizers, which satisfies current tea industry objectives (Liu et al., 2023b).

### 5.4 Irrigation regulation

Irrigation is the primary method for regulating the water supply during commercial tea production because it can optimize soil moisture conditions to ensure high-quality leaves are produced. Irrigation practices can be classified as drip irrigation and sprinkler irrigation. Drip irrigation emitters direct water to root-dense zones and maintain soil moisture levels at an optimal proportion of field capacity. This stabilizes leaf stomatal conductance at a favorable level, which prevents photosynthetic inhibition and enhances water use efficiency, while also improving soil physicochemical properties, promoting nutrient absorption, and ensuring the balanced accumulation of polyphenols and amino acids to maintain consistent quality (Chen et al., 2010; Liu, 2022). Suitable for hilly terrains and water-deficient tea gardens, drip irrigation may be combined with soil moisture monitoring to develop methods tailored to specific conditions. Sprinkler irrigation improves microclimatic conditions through atomized spraying, leading to increased canopy humidity, which can mitigate the harmful effects of high-temperature stress and decrease leaf oxidative damage. The uniformity of water distribution by sprinkler irrigation systems can promote the uniform emergence of new shoots and leaf area expansion, while inducing a noticeable increase in leaf chlorophyll and carotenoid contents respectively. Moreover, net photosynthetic rates may surpass those of traditional flood irrigation systems by a considerable margin, while amino acid and polyphenol accumulation is modulated to optimize quality (Zou et al., 2022). Furthermore, sprinkler irrigation is suitable for large tea plantations on plains and in hot and arid regions. Although drip irrigation and sprinkler irrigation have specific uses, topography, climate, and cultivar traits must be considered before selecting an irrigation system. They may also be combined with fertilization practices to optimize water and nutrient availability for leaf growth.

### 5.5 Shade regulation

As an artificial cultivation technique with regulatory effects on tea leaf development, shading involving different shade net materials (e.g., red, blue, silver-gray, and black) and a specific shading intensity and duration may enhance tea plant growth (Ge et al., 2024; Elango et al., 2023). Shading primarily alters light conditions (e.g., decreased light intensity and optimized light quality) to influence leaf development. At the physiological level, shading protects photosynthetic systems, enhances photosynthesis, increases chlorophyll and carotenoid contents (Elango et al., 2023; Chen et al., 2021c), regulates hormone synthesis and lignin biosynthesis (Teng et al., 2021; Fang et al., 2022), and alleviates heat stress due to intense sunlight in summer (Ge et al., 2024). In terms of its effects on plant morphology and yield, shading increases the leaf area, new shoot bud density, and leaf tenderness (Ge et al., 2024; Fang et al., 2022). It also can modulate leaf quality-related metabolism by increasing amino acid contents, optimizing the phenol-to-amino acid ratio, and controlling the accumulation of metabolites associated with tea quality (Ge et al., 2024; Elango et al., 2023). Colored shade nets generally outperform black shade nets in terms of overall efficacy, but appropriate shading parameters must be selected to avoid potential problems, including decreased leaf stress tolerance (Ge et al., 2024). Plantations growing tea varieties for matcha tea production use a standardized shading system, which represents the most common shading system used for tea cultivation. More specifically, black shade nets (1.5 m height and 70% shading rate) are used for shading at approximately 30 days before harvest. This shading can optimize temperature and light conditions and promote chlorophyll synthesis, while significantly increasing amino acid contents and decreasing polyphenol contents. Accordingly, leaf quality requirements for matcha tea production (vibrant emerald green color, fresh and brisk taste, and low astringency) may be achieved using this shading system (Wu et al., 2023).

## 6 Concluding remarks and future perspectives

Multi-dimensional mechanisms regulating tea leaf development are presented herein. More specifically, tea quality and yield depend on leaf development synergistically regulated by intrinsic genetic programs and environmental factors. Morphogenesis follows a three-stage model: primordial initiation, primary morphogenesis and expansion, and secondary morphogenesis. Tea leaves have adapted specifically to subtropical environments (e.g., sunken stomata and well-developed palisade tissues). Endogenous regulatory mechanisms mainly involve hormones and transcription factors, with growth-promoting hormones, such as IAA, GA, and CTK, controlling cell division and expansion. By contrast, stress-responsive hormones, including ABA, mediate stress resistance-related processes, while transcription factors, including WRKY, bHLH, TCP, and MYB family members, precisely regulate leaf development, secondary metabolism, and stress responses through intricate networks. Natural environmental factors, such as light, temperature, moisture, altitude, and pests/diseases, substantially influence leaf morphological, physiological, and metabolic characteristics via endogenous signaling pathways. Furthermore, agronomic practices, including intercropping, pruning, fertilization, irrigation, and shading, markedly affect tea leaf development and quality. The insights provided in this review may be relevant to filling knowledge gaps regarding the multi-dimensional regulation of tea leaf development. Information provided herein may serve as reference material for future in-depth research on key gene functions, multi-factor interactions, and mechanisms underlying the synergism between environmental and anthropogenic effects. This review offers a robust theoretical foundation for improving tea cultivars and developing efficient cultivation practices.

To gain deeper insights into the regulatory networks governing tea leaf development and precisely control these networks during the commercial cultivation of tea plants, future research should focus on the following. First, multi-omics research should be integrated with systems biology-related analyses (Kundu and Tanti, 2025; Zhao et al., 2025a). Current research in this field predominantly involves functional analyses of specific genes, future work must integrate multi-dimensional data (e.g., genome, transcriptome, proteome, metabolome, and epigenome) to construct dynamic regulatory network maps of tea leaf development. Clarifying the interactions and spatiotemporal specificity of the “hormone–transcription factor–target gene” module across developmental stages and under environmental stress conditions should be prioritized. On the basis of single-cell sequencing and other molecular techniques, we may be able to reveal the molecular basis of leaf tissue differentiation and functional specialization at the cellular level, thereby systematically elucidating complete regulatory pathways from genes to traits. Unfortunately, a gene functional verification system has not been established for tea. Specifically, there is an urgent need for an efficient genetic transformation system, such as the CRISPR-Cas12i gene-editing tool, that can be used to systematically verify the functions of candidate tea genes, including *CsTCP2* and *CsMYB1* (Huo et al., 2025). Second, research on the synergistic mechanisms of multi-dimensional regulatory factors should be emphasized (Zhu et al., 2025). Leaf development is influenced by the combined effects of genetic programs, environmental signals, and cultivation methods. Future research should transcend single-factor studies to: (1) investigate cross-talk mechanisms among key endogenous signals (e.g., between hormones and between hormones and transcription factors); (2) elucidate how environmental factors (e.g., light and temperature stress) reprogram endogenous networks via epigenetic modifications and non-coding RNAs; (3) reveal the interactive regulatory effects of agronomic practices (e.g., pruning and fertilization), endogenous tea plant developmental mechanisms, and environmental conditions. This research will depend on the development and/or use of genetic transformation systems and gene-editing tools for tea plants to functionally validate and manipulate core regulatory nodes; and (4) construct an integrated hormone metabolism model on the basis of model plants, while also establishing a dynamic regulatory network map of tea leaf development (Fàbregas et al., 2026). Finally, research outcomes should be exploited to design intelligent cultivation and breeding practices. The ultimate goal of fundamental research is to provide guidance for practical applications (Li et al., 2025). Thus, future studies should aim to: (1) leverage identified key genes to improve tea quality, stress tolerance, and light use efficiency through marker-assisted selection and gene-editing technologies to develop new varieties tailored to diverse ecological and production requirements. (2) construct dynamic models linking leaf developmental states with environmental and management factors, while integrating sensor monitoring and artificial intelligence analyses to achieve real-time perception and intelligent decision-making for light, temperature, water, and fertilizer management in tea gardens, thereby developing “predictable and controllable” cultivation models for enhancing leaf quality, and (3) improve sustainable tea garden management systems designed to regulate leaf development (e.g., application of environmentally friendly pest control and cultivation practices). In summary, on the basis of cross-scale integration, multi-dimensional interactive research, and industry–academia collaborations, we aim to comprehensively decipher the tea leaf development, thereby providing a foundation for high-quality, efficient, environmentally safe, and intelligent improvements to the tea industry.
